# Nonlethal, Epigenetic Age Estimation in a Freshwater Sportfish, Florida Bass (*Micropterus salmoides*)

**DOI:** 10.1002/ece3.72495

**Published:** 2025-11-10

**Authors:** D. Nick Weber, Summer Lindelien, Andrew C. Dutterer, Jason R. Dotson, Jennifer Moran, Andrew T. Fields, William F. Patterson, David S. Portnoy

**Affiliations:** ^1^ Marine Genomics Laboratory Texas A&M University‐Corpus Christi Corpus Christi Texas USA; ^2^ Gainesville Freshwater Fisheries Office, Fish and Wildlife Research Institute, Florida Fish and Wildlife Conservation Commission Gainesville Florida USA; ^3^ Eustis Fisheries Research Laboratory Fish and Wildlife Research Institute, Florida Fish and Wildlife Conservation Commission Eustis Florida USA; ^4^ School of Forest, Fisheries, and Geomatics Sciences University of Florida Gainesville Florida USA

**Keywords:** black bass, DNA methylation, epigenetic clock, nonlethal age estimation

## Abstract

Black basses (*Micropterus* spp.) are the most popular freshwater sportfishes in North America, with high recreational, societal, and economic value. The conservation and management of black bass fisheries rely on the generation of age data to estimate population dynamics. Age data are typically produced via counts of opaque zones in otolith sections, a necessarily lethal process. The development of an accurate, nonlethal age estimation method would expand opportunities for collecting age‐related information in cases where sacrificing fish is either not an option (e.g., tournaments) or undesirable (e.g., trophy‐sized fish). An epigenetic clock was developed for Florida bass ( *Micropterus salmoides* ) using DNA extracted from fin clips and enzymatically converted restriction site‐associated DNA sequencing (EC‐RADseq). CpG sites exhibiting age‐correlated DNA methylation were identified using Bayesian generalized linear models in individuals (*n* = 176; age range: 0.84–13.21 years) captured from six different water bodies across Florida, USA, and potential environmentally influenced, age‐predictive sites were identified and removed. The epigenetic clock developed using elastic net regression was highly accurate (mean absolute error, MAE = 0.28 years) and precise (*R* ^2^ = 0.98). Overall, results demonstrate an accurate, nonlethal alternative to otolith‐based age determination of Florida bass and could likely be applied to other black basses. In addition, when compared to traditional age estimation via hard structures, epigenetic age estimation is relatively rapid and cost‐effective, with important implications for black bass assessment, management, and conservation.

## Graphical abstract

The conservation and management of black bass fisheries rely on the generation of age data to estimate population dynamics, and the development of an accurate, nonlethal age estimation method would expand opportunities for collecting age‐related information in cases where sacrificing fish is either not an option or undesirable. Herein, an epigenetic clock was developed for Florida bass ( *Micropterus salmoides* ) using DNA extracted from fin clips and enzymatically‐converted restriction site‐associated DNA sequencing (EC‐RADseq). Overall, results demonstrate an accurate, nonlethal alternative to otolith‐based age determination of Florida bass, and could likely be applied to other black basses, with important implications for black bass assessment, management, and conservation.

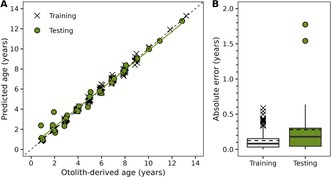

## 1 Introduction

Black basses (*Micropterus* spp.) are the most popular freshwater sportfishes in North America, with high recreational, societal, and economic value (Siepker et al. 2007). As such, black bass are intensively managed by natural resource agencies, and accurate and precise age data are required for the estimation of population dynamics (e.g., recruitment, growth, mortality; Griffin et al. 2022). Otoliths are the preferred aging structure for many fish species, because otolith‐based ages tend to be more accurate and precise than ages generated from other aging structures (Phelps et al. 2017). However, the generation of otolith‐based ages is costly and time‐intensive (Helser et al. 2019), and the removal of otoliths is a necessarily lethal process. This is particularly problematic when sacrificing fish is either not an option (e.g., live release tournaments) or undesirable during routine monitoring (e.g., trophy‐sized fish; Lindelien et al. 2024). Thus, the development of an accurate, nonlethal aging approach would be of great utility for black bass population assessment and management and would allow for avenues of research rendered infeasible by the lethality of otolith‐based age estimation (e.g., understanding the effects of environmental stress on longevity and growth).

Nonlethal aging via alternative hard structures has been explored for black bass species (e.g., using fin rays and fin spines; Griffin et al. 2022; Lindelien et al. 2024) but has lower precision and accuracy as compared to otolith‐based aging. DNA methylation‐based, epigenetic aging techniques offer an alternative nonlethal aging approach, potentially without compromising aging accuracy or precision (Weber, Fields, et al. 2024). Epigenetic aging techniques have generated increasing interest in recent years, due in large part to the fact that they are nonlethal, inexpensive relative to traditional age estimation using hard structures, and well‐suited for rapid, large‐scale age estimation (Mayne et al. 2023). Epigenetic aging is made possible by the process of DNA methylation, which refers primarily to the addition of methyl groups (CH_3_) to cytosines located within CpG dinucleotides (cytosines followed by guanines; Moore et al. 2013). Changes in DNA methylation levels at certain CpG sites correlate with chronological age, which enables the development of age‐predictive models based on changes in DNA methylation, referred to as epigenetic clocks (Anastasiadi and Piferrer 2023).

The utility of epigenetic age estimation has been demonstrated in a handful of teleost species (reviewed in Piferrer and Anastasiadi 2023; Mayne et al. 2023; Weber, Fields, et al. 2024), but the technique has not been widely applied in freshwater systems. Freshwater systems differ from marine systems in that they are often characterized by a higher degree of environmental variation over smaller spatial and temporal scales (Arnosti et al. 2014). While changes in DNA methylation can be environmentally induced (Angers et al. 2010), the degree to which environmental variation affects epigenetic clock performance remains poorly understood (Piferrer and Anastasiadi 2023). To this end, accounting for potential environmentally influenced sites (i.e., sites for which methylation levels depend on the magnitude of an environmental factor; Garg et al. 2018), during epigenetic clock development may be necessary to maximize clock performance.

Florida bass ( *Micropterus salmoides* ) have a natural range from southern Florida to the Carolinas in the USA (Kim et al. 2022) and represent a large component of the targeted catch in the freshwater sportfishing industry, due in large part to their fast growth rates and relatively large maximum body size (Barthel et al. 2010). As with other black basses, Florida bass are traditionally aged via counts of opaque zones in otoliths, a technique that has been validated in the species to age five (Hoyer et al. 1985). Because individuals that attain trophy size (e.g., ≥ 3.6 kg) are rarely encountered in targeted sampling, retaining those individuals in the population is desirable (Lindelien et al. 2024). In addition, Florida bass management efforts include encouraging recreational anglers to release trophy‐sized fish to allow for future catch‐and‐release opportunities (Dutterer et al. 2014). Consequently, there is an inability to collect age‐related information about the largest individuals, which may lead to biased estimates of population parameters (Lindelien et al. 2024). The ability to accurately, nonlethally estimate age would benefit black bass assessment and management by allowing fisheries scientists to estimate age across individuals of all sizes, thereby reducing bias in population parameter estimates without sacrificing trophy‐sized bass. Therefore, the goal of the present study was to develop an epigenetic clock for Florida bass captured from six different water bodies across Florida. The objectives of the present study were to: (1) identify CpG sites exhibiting age‐correlated DNA methylation across the Florida bass genome; (2) identify and remove potential environmentally influenced, age‐correlated CpG sites; and (3) construct an age‐predictive model (epigenetic clock) relating levels of DNA methylation at selected CpG sites to otolith‐derived age.

## 2 Methods

### 2.1 Sample Collection

**FIGURE 1:**
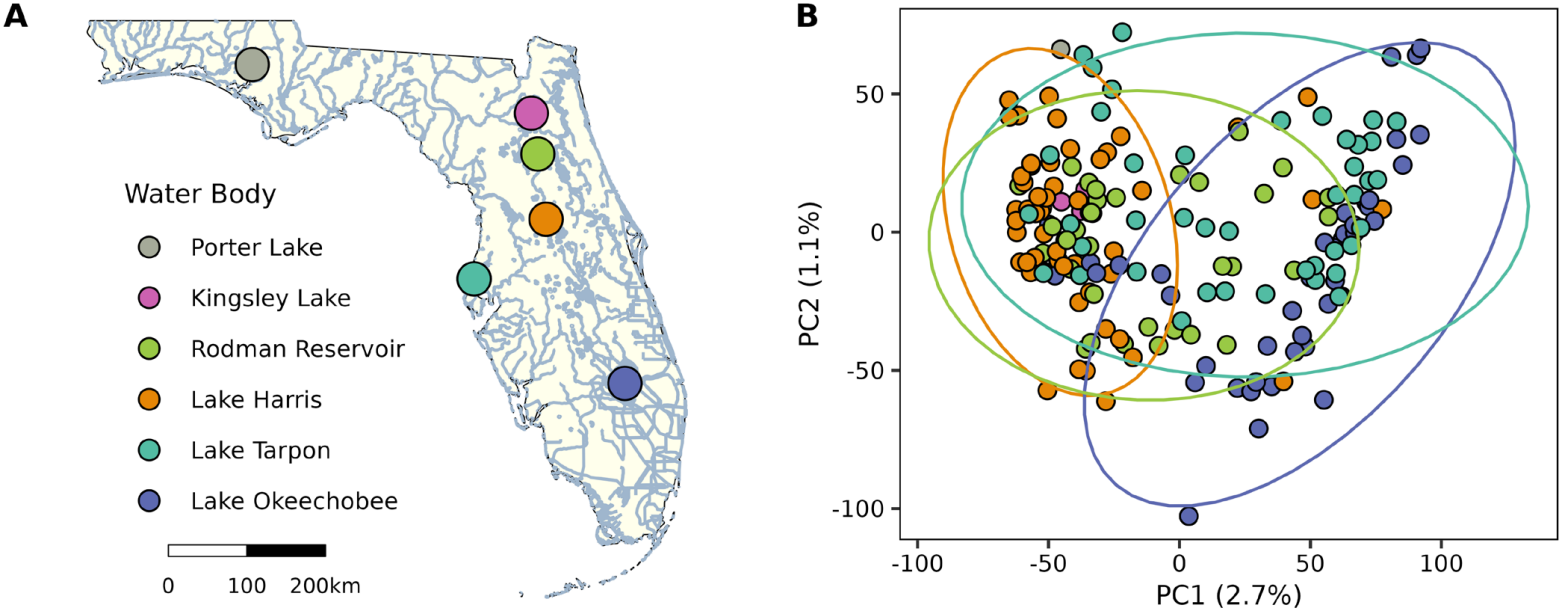
Panel (A) indicates Florida bass ( *Micropterus salmoides* ) sample collection locations across the state of Florida, USA. Panel (B) depicts a principal component analysis (PCA) of percent methylation across CpG sites (*n* = 118,349) with 95% confidence ellipses and colors corresponding to the key in (A).

Otoliths and fin clips were collected from Florida bass (*n* = 1045) caught between 2023 and 2024 in six different water bodies (Porter Lake, Kingsley Lake, Rodman Reservoir, Lake Harris, Lake Tarpon, Lake Okeechobee) across Florida (Figure 1A). Fish were captured using daytime boat electrofishing, hook‐and‐line, or were recovered as carcasses, weighed, and measured to the nearest mm total length (TL). Sagittal otoliths were extracted from fish, cleaned, and air‐dried, while fin clips (approximately 0.5 cm^2^) were immersed in 20% DMSO–0.25 M EDTA NaCl‐saturated buffer (Seutin et al. 1991) and stored at room temperature until DNA extraction. All sampling was conducted by the Florida Fish and Wildlife Conservation Commission (FWC) in accordance with the Guidelines for the Use of Fishes in Research (Use of Fishes in Research Committee; Joint Committee of the American Fisheries Society, the American Institute of Fishery Research Biologists, and the American Society of Ichthyologists and Herpetologists 2014) and was reviewed and approved by Florida Fish and Wildlife Research Institute administration.

Fish integer age was estimated by two independent readers via opaque zone counts in thin cross‐sections of otoliths using the protocol described in Lindelien et al. (2024), and a subset of individuals (*n* = 180) for which both readers agreed on the age were selected for epigenetic clock development. Subsequently, both readers recounted opaque zones in this subset of individuals to assess intra‐observer repeatability. Mean intra‐observer error was calculated by dividing the number of disagreements between original and recount estimates by the total number of recounts for each reader and then calculating the mean among observers (Gunn et al. 2025). For these individuals, fractional age was calculated by dividing the sum of days alive among integer years and days alive in the last year by 365. Days alive among integer years was equal to the number of opaque zones multiplied by 365. Days alive in the last year was equal to the number of days surpassed between the collection date and March 15. March 15 was the assumed median birth date based on the peak reproductive period (January through April) for Florida bass in central Florida lakes (Rogers and Allen 2009).

### 2.2 Genomic Library Preparation

Genomic DNA was extracted from fin clips using the Mag–Bind Blood and Tissue DNA Kit (Omega Bio‐tek Inc., Norcross, USA), and two libraries were prepared for enzymatically‐converted restriction site‐associated DNA sequencing (EC‐RADseq). Briefly, restriction digests were performed using *Mfe*I‐HF, and unique hemi‐methylated barcoded adaptors were ligated to each sample (Peterson et al. 2012). Samples were then pooled, sheared (Covaris M220 Ultrasonicator; Covaris Inc., Woburn, USA), and size selected using a Pippin Prep Size Selection System (Sage Science Inc., Beverly, USA). Libraries were then split into two portions, and one portion was treated with a methylation‐sensitive enzyme using the NEBNext Enzymatic Methyl‐seq Kit (New England Biolabs, Ipswich, USA). This enzyme treatment converts unmethylated cytosines into uracils through chemical deamination, and uracils are subsequently replaced by thymines during PCR. This results in predictable base substitutions at all unmethylated cytosines, which can be identified by comparing sequences from the treated portion to the untreated portion. The libraries (including both the treated and untreated portions) were then sequenced across two lanes on an Illumina NovaSeq 6000 (Illumina Inc., San Diego, USA). Reads were mapped to the *M. salmoides* genome from NCBI (GenBank: JAKUMD000000000.1; He et al. 2022), and mapped reads were filtered to retain primary alignments, proper pairs, and those with a mapping quality ≥ 40.

### 2.3 Quality Control and Data Filtering

CpG sites that could not be successfully genotyped in the untreated portion or that were identified as potential single nucleotide polymorphisms (SNPs; defined as sites where > 5% of total untreated reads across individuals displayed a cytosine to thymine substitution on the forward strand or guanine to adenine substitution on the reverse strand; Weber et al. 2022; Weber, Fields, et al. 2024; Weber, Wyffels, et al. 2024) were removed from the dataset. Individuals with low sequencing coverage (defined by the presence of < 400,000 CpG sites) were also removed from the dataset. CpG sites were then filtered to retain only sites present in ≥ 80% of individuals. Percent methylation was estimated as the number of methylated reads divided by the total number of reads, and per‐site 95% confidence intervals were calculated around the estimate for each individual (Clopper and Pearson 1934). Only those sites with confidence intervals < 0.85 in at least 80% of individuals were retained, which was roughly equivalent to a mean of 16 reads per site (Weber et al. 2022; Weber, Fields, et al. 2024; Weber, Wyffels, et al. 2024). Principal component analysis (PCA) was then conducted using the package *FactoMineR* version 2.11 on percent methylation across CpG sites to visually assess for potential environmental signal (i.e., separation by water body of capture).

### 2.4 Identification of Age‐Correlated DNA Methylation

To identify CpG sites exhibiting age‐correlated DNA methylation, a Bayesian framework was used to estimate the parameters of a generalized linear model (GLM) using the package *rstanarm* version 2.19.3 (Goodrich et al. 2020). For each CpG site, the odds ratio of the number of methylated reads (Meth) to unmethylated reads (Total − Meth) was modeled as a linear function of age:

(1) $$\frac{\text{Meth}}{\text{Total}-\text{Meth}}=e^{\left(\beta_{0}+\beta_{1}\mathrm{Age}+\mu_{\left[\text{Fish}\right]}\right)}$$

where *β*_0_ represents the overall intercept; *β*_1_ represents the fixed effect of otolith‐derived fish age; and *μ*_[Fish]_ represents a random intercept for individual fish, with $\mu_{\left[\text{Fish}\right]}\sim N\left(0,\sigma_{\text{Fish}}^{2}\right)$. The default, weakly informative priors in *rstanarm* were used, and posterior distributions were estimated with four independent Markov Chain Monte Carlo (MCMC) runs, each run for 4000 iterations. Runs were considered to have converged if the effective sample size (*n*_eff) was greater than 2000 and the Gelman‐Rubin convergence diagnostic was less than 1.01 (Lunn et al. 2013; Muth et al. 2018). Sites with a 95% credible interval that did not include zero for the slope of otolith‐derived age versus methylation were considered to exhibit significant age‐correlated methylation. For each CpG site that exhibited age‐correlated methylation, individuals with over‐dispersed confidence intervals (> 0.85) were entered as missing data. Because downstream analysis does not allow for missing data, methylation frequencies at missing sites for a given individual were imputed as the median value of percent methylation present at that site in individuals of the same (or most similar) otolith‐derived age.

### 2.5 Removal of Age‐by‐Environment Signal

PCA was used to visually assess for the potential presence of environmentally influenced, age‐predictive CpG sites, and permutational analysis of variance (PERMANOVA) with 9999 permutations was used to assess for significant differentiation between groups observed on the PCA at an alpha level of 0.05. To remove the observed environmental signal, the dataset was subset to include only Lake Harris and Lake Okeechobee, and discriminant analysis of principal components (DAPC, performed using *adegenet*, version 2.1.10; Jombart 2008) with *K*‐means clustering was performed for *K* = 2 and validated using 50 repetitions at each level of PCs retained for a maximum of 300 PCs. The variables (CpG sites) contributing the most to the first discriminant function were then iteratively removed from the dataset until the water bodies of interest visually collapsed into one group on the PCA, and the PERMANOVA was then repeated to confirm removal of the environmental signal.

### 2.6 Epigenetic Clock Construction

The relationship between otolith‐derived age and percent methylation across the remaining CpG sites exhibiting age‐correlated methylation was characterized using elastic net penalized regression modeling, as implemented in the package *glmnet* version 4.1.8 (Friedman et al. 2010). Elastic net regression is a regularization technique that combines the strengths of lasso and ridge regression and is particularly useful when the number of predictor variables (i.e., CpG sites) is much bigger than the number of observations (i.e., individuals; Zou and Hastie 2005). Consequently, elastic net regression has become the standard method for the de novo development of epigenetic clocks (Tomo and Nakaki 2024). Prior to penalized regression modeling, 80% of samples were randomly assigned to a training dataset and the remainder into a testing dataset, using the “partition” function from the package *groupdata2* version 2.0.3. Despite the relative strengths of elastic net regression in handling large numbers of predictor variables (Zou and Hastie 2005), elastic net regression tends to over‐select features and thus requires a data reduction step (Liu et al. 2023). To reduce the volume of data going into modeling and thus the probability of over‐selection, independent Pearson correlation coefficients between otolith‐derived age and percent methylation among individuals in the training dataset were calculated for each CpG site (Bertucci et al. 2021), using the function “corr.test” from the package *psych* version 2.4.3. Both the training and testing datasets were then subset for the CpG sites with the largest 1000 absolute correlation coefficients, and otolith‐derived age was natural log‐transformed (Mayne et al. 2020, 2021). To identify the subset of sites which minimized mean absolute error (MAE) in the final predictive model, a custom R script (available at https://github.com/marinegenomicslab/FloridaBass_EpigeneticAgeing) was used to randomly select 125 CpG sites with replacement over 600,000 iterations and run the “cva.glmnet” function, which simultaneously cross validates both the penalty and alpha parameters.

Performance of the final predictive model was assessed using linear regression and by computing median absolute error (MDAE) and mean absolute error (MAE) between otolith‐derived age and age predicted by the epigenetic clock. Relative error was calculated by dividing the absolute error by the otolith‐derived age, and linear regression was conducted on otolith‐derived age versus relative error to determine if error in the model increased or decreased with increasing age. All analyses were conducted in *R* version 4.4.3 (R Core Team 2025).

## 3 Results

### 3.1 Dataset Summary

Florida bass (*n* = 1045) were captured from six different water bodies across Florida (Figure 1A), and otolith‐based age estimates were highly precise (index of average percent, iAPE = 0.31%). Of these, a subset of individuals (*n* = 180) for which both readers agreed on the otolith‐based age estimate (i.e., iAPE = 0%) and which spanned the available age range (0.84–13.21 years) were selected for epigenetic clock development. Average intra‐observer error across the selected individuals was 0.003.

A total of 4.24 billion paired, raw reads were obtained (mean = 17.52 million reads per individual). After filtering to retain primary alignments, proper pairs, and those with a mapping quality ≥ 40, an average of 74.0% of reads per individual mapped to the *M. salmoides* genome. A total of 3,404,777 CpG sites were recovered, of which 243,109 sites either could not be successfully genotyped in the untreated portion of the library or were identified as potential SNPs and removed from the dataset. Additionally, four individuals were removed due to low sequencing coverage (i.e., < 400,000 CpG sites per individual). In total, 182,668 CpG sites were present in > 80% of individuals, of which 64,319 were removed due to excessively wide confidence intervals in > 20% of individuals. The final, filtered dataset contained 176 individuals and 118,349 CpG sites and spanned a fractional, otolith‐derived age range of 0.84–13.21 years (Table 1). Mean (±SD) global CpG methylation across the retained sites was 80.36% (±1.04%).

**TABLE 1:** Sample summary and morphometrics for Florida bass ( *Micropterus salmoides* ) included in epigenetic clock development and testing.

Water body	*n*	Integer age (years)	Fractional age (years)	Total length (mm)	Weight (g)
Lake Harris	54	1–10	0.89–10.00	148–625	28–3603
Lake Okeechobee	42	1–12	0.84–11.80	194–591	97–3712
Lake Tarpon	42	1–9	0.98–8.98	178–558	71–2545
Rodman Reservoir	34	1–9	0.99–9.05	152–623	39–3648
Kingsley Lake	3	9–13	8.87–13.21	493–640	1175 − 3498
Porter Lake	1	11	11.00	615	3266

### 3.2 Identification of Age‐Correlated Methylation and Removal of Age‐by‐Environment Signal

**FIGURE 2:**
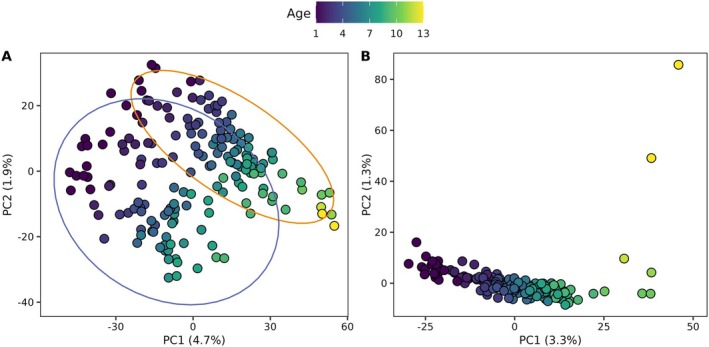
Principal component analysis (PCA) of percent methylation across CpG sites identified as exhibiting significant age‐correlated DNA methylation. Panel (A) includes all CpG sites exhibiting age‐correlated methylation (*n* = 10,899), with 95% confidence ellipses around the individuals from Lake Harris (orange) and Lake Okeechobee (purple). Panel (B) includes the CpG sites (*n* = 5450) retained after removing environmentally influenced, age‐predictive sites via discriminant analysis of principal components (DAPC).

Principal component analysis of percent methylation indicated separation between individuals from Lake Harris and Lake Okeechobee (Figure 1B). Bayesian GLMs identified 10,899 CpG sites that exhibited significant age‐correlated methylation, with significant differentiation between individuals from Lake Harris and Lake Okeechobee (PERMANOVA, pseudo‐*F* = 2.96, *R* ^2^ = 0.03, *P* < 0.001; Figure 2A). Cross‐validation for DAPC (*K* = 2) returned 96% assignment for the two groups (i.e., water bodies) with 10 PCs retained. The top 50% of variables (CpG sites) contributing to the first discriminant function were removed from the dataset, leaving 5450 CpG sites that exhibited significant age‐correlated methylation, with no visual separation among water bodies and no differentiation between individuals from Lake Harris and Lake Okeechobee (PERMANOVA, pseudo‐*F* = 1.03, *R* ^2^ = 0.01, *P* = 0.222; Figure 2B).

### 3.3 Epigenetic Clock Performance

**FIGURE 3:**
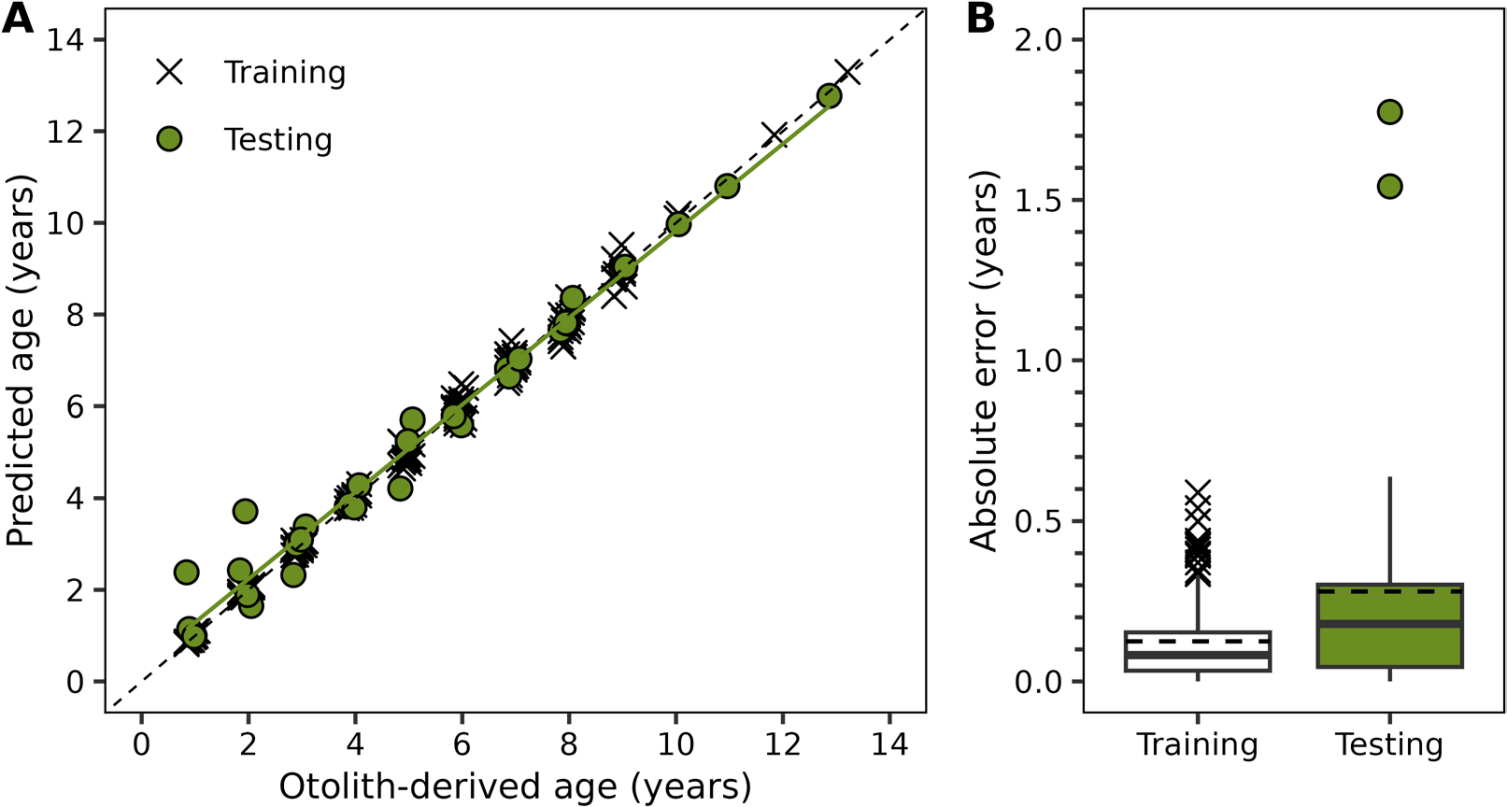
Epigenetic clock developed for Florida bass ( *Micropterus salmoides* ). Panel (A) depicts epigenetic age predictions versus otolith‐derived ages for both the training and testing datasets. Dashed line indicates line of 1:1 agreement between predicted and otolith‐derived age. Solid line represents a linear regression fit to the data. Panel (B) depicts absolute error for both the training and testing datasets. Solid lines in boxplots represent median absolute error (MDAE), and dashed lines represent mean absolute error (MAE).

Penalized regression analysis selected 123 CpG sites in the final, age‐predictive model. Strong agreement was observed between otolith‐derived age and predicted age in both the training (*R* ^2^ = 0.99) and testing (*R* ^2^ = 0.98) datasets (Figure 3A; Table 2). Median absolute error (MDAE) was 0.08 and 0.18 years in the training and testing datasets, respectively (Figure 3B; Table 2). Mean absolute error (MAE) was 0.13 and 0.28 years in the training and testing datasets, respectively (Figure 3B; Table 2). Relative error in the testing dataset decreased with increasing age (*P* = 0.02; *R* ^2^ = 0.17).

**TABLE 2:** Summary statistics for the epigenetic clock developed for Florida bass ( *Micropterus salmoides* ), including the number and age range of individuals used, the coefficient of determination (*R* ^2^), the median absolute error (MDAE) reported in years, and the mean absolute error (MAE) reported in years.

Dataset	*n*	Fractional age (years)	*R* ^2^	MDAE (years)	MAE (years)
Training	141	0.84–13.21	0.99	0.08	0.13
Testing	35	0.84–12.87	0.98	0.18	0.28

## 4 Discussion

The development of an epigenetic clock for Florida bass offers an effective, nonlethal alternative to otolith‐based age estimation. The high accuracy and precision reported are particularly important in the context of fisheries assessment and management, given that error in age estimation propagates as bias or imprecision in the estimation of population parameters (e.g., growth, mortality, yield per recruit; Lai and Gunderson 1987). Moreover, the ability to accurately, nonlethally estimate age will enable age estimation in scenarios in which sacrificing fish is undesired (e.g., small populations, populations closed to harvest, and populations intensively managed for trophy‐sized fish; Lindelien et al. 2024).

Nonlethal age estimation of Florida bass using fin rays and spines has been investigated in recent years, but these structures suffer from decreased precision when compared to the use of otoliths (Lindelien et al. 2024). For instance, among the individuals included in that study, the index of average percent error (iAPE) for otolith‐based age estimates was 0.97% but ranged from 3.20% to 5.97% for age estimates derived from fin structures (see supplementary tables in Lindelien et al. 2024). In addition, bias varied among readers, suggesting that reader experience or skill may influence the utility of fin structure age estimation. To this end, the epigenetic clock developed for Florida bass in the present study was not only highly accurate and precise, but epigenetic age estimation benefits from the removal of any potential reader bias, as no readers are involved.

Model performance increased with increasing age, which is contrary to previously *de novo* developed epigenetic clocks for fishes (e.g., Weber, Fields, et al. 2024; Weber, Wyffels, et al. 2024), in which model performance did not change with increasing age. In addition, this result is contrary to epigenetic clocks developed in previous studies which applied conserved age‐associated CpG sites identified in the zebrafish ( *Danio rerio* ; Mayne et al. 2020) to other species, which suffer from decreasing accuracy with increasing age (e.g., Mayne et al. 2021, 2023). While not yet documented in fishes, the increased error observed at younger ages could be a result of more rapid and dynamic changes in DNA methylation in juveniles—a phenomenon that is well described in the human epigenetic aging literature (Alisch et al. 2012) and attributed to the decreased performance of human epigenetic clocks in younger individuals (McEwen et al. 2019). Alternatively, it is possible that the Bayesian GLMs and elastic net regression models used to build the epigenetic clock did not capture the most informative CpG sites for younger individuals, and that the epigenetic clock constructed could be improved if such sites were to be found and included. Nonetheless, the high accuracy observed at older ages is particularly important for the ability to obtain accurate age estimates for some of the largest and oldest fish in the context of trophy bass research and management.

The degree to which environmental variation can affect epigenetic clock performance remains poorly understood (Piferrer and Anastasiadi 2023), and the limited, currently available literature is contradictory. Elevated water temperatures had no effect on age prediction in aquarium‐reared, juvenile European sea bass ( *Dicentrarchus labrax* ), suggesting epigenetic clocks can predict age independently of some environmental variation (Anastasiadi and Piferrer 2020). However, ionizing radiation changed the rate of DNA methylation at age‐associated CpG sites in aquarium‐reared medaka ( *Oryzias latipes* ) and influenced epigenetic clock performance (Bertucci et al. 2021). In the present study, PCA results suggested the presence of an age‐by‐environment signal across the water bodies sampled, and DAPC was used to remove sites where environmental influence might obscure age‐related patterns, thus maximizing the performance of the epigenetic clock across all water bodies. Therefore, while the potential effects of including environmentally influenced sites in epigenetic clock development remain unknown, results presented suggest highly accurate and precise epigenetic clocks can be developed by identifying and removing such sites. Future research related to the degree of influence that environmental factors may have on epigenetic clock performance, and the types of environmental factors driving this influence (e.g., water temperature, water quality, food availability) is warranted.

The results presented for Florida bass demonstrate accurate and precise species‐specific epigenetic clocks can be developed for other black basses (*Micropterus* spp.), for which age estimates have also suffered from decreased accuracy and precision when using nonlethally sampled hard structures (e.g., largemouth bass, *M. nigricans* ; smallmouth bass, *M. dolomieu* ; spotted bass, *M. punctulatus* ; Klein et al. 2017; Blackwell et al. 2019; Griffin et al. 2022). Moreover, if the CpG sites included in the epigenetic clock developed for Florida bass are conserved between Florida bass and other *Micropterus* species, it is possible that the epigenetic clock developed could be applied to other *Micropterus* species and would eliminate the need to build additional species‐specific clocks for the genus. This would be particularly important for *Micropterus* species of greatest conservation need (e.g., shoal bass *Micropterus cataractae*, Henry et al. 2024) and threatened *Micropterus* species (e.g., Bartram's bass *Micropterus* sp. cf. *cataractae*, Taylor et al. 2019). However, the extent to which CpG sites exhibiting age‐correlated DNA methylation are conserved between both closely and distantly related fish species remains largely unknown. Some of the 29 CpG sites included in an epigenetic clock developed for zebrafish ( *Danio rerio* ; Mayne et al. 2020) have been shown to be conserved across a range of species (e.g., Australian lungfish *Neoceratodus forsteri* , Murray cod *Maccullochella peelii* , Mary River cod *Macculluchella mariensis*, golden perch *Macquaria ambigua* ; Mayne et al. 2021, 2023), but epigenetic clocks developed using those sites have been characterized by varying levels of performance. The degree to which CpG sites exhibiting age‐correlated DNA methylation are conserved between both closely and distantly related species, and the potential development of multi‐species epigenetic clocks with levels of accuracy adequate for fisheries assessment, remain important areas of future research.

## 5 Conclusions

Overall, the present study adds to a growing body of literature on the development and application of epigenetic clocks for fishes (reviewed in Anastasiadi and Piferrer 2023; Piferrer and Anastasiadi 2023). The results presented for Florida bass indicate accurate and precise epigenetic clocks can be developed for black basses (*Micropterus* spp.), which would enable age estimation in scenarios in which sacrificing fish is undesired (e.g., small populations, populations closed to harvest, and populations intensively managed for trophy‐sized fish; Lindelien et al. 2024). In addition, when compared to traditional aging techniques, the use of epigenetic clocks to obtain age estimates is relatively rapid, inexpensive (Mayne et al. 2021), and not hindered by between‐reader bias. Finally, this study demonstrates the identification and removal of potential environmentally influenced CpG sites, with important implications for the development of epigenetic clocks in species that range across heterogeneous environments.

## Author Contributions

**D. Nick Weber:** conceptualization (equal), data curation (equal), formal analysis (lead), methodology (lead), software (lead), writing – original draft (lead), writing – review and editing (equal). **Summer Lindelien:** conceptualization (equal), data curation (equal), funding acquisition (equal), investigation (equal), writing – review and editing (equal). **Andrew C. Dutterer:** conceptualization (equal), data curation (supporting), funding acquisition (equal), investigation (supporting), writing – review and editing (equal). **Jason R. Dotson:** conceptualization (equal), funding acquisition (lead), investigation (supporting), writing – review and editing (equal). **Jennifer Moran:** data curation (supporting), resources (supporting), writing – review and editing (equal). **Andrew T. Fields:** formal analysis (supporting), methodology (supporting), software (supporting), writing – review and editing (equal). **William F. Patterson III:** conceptualization (equal), investigation (supporting), writing – review and editing (equal). **David S. Portnoy:** conceptualization (equal), investigation (supporting), resources (lead), supervision (lead), writing – review and editing (equal).

## Conflicts of Interest

The authors declare no conflicts of interest.

## Data Availability

Datasets and data analysis scripts are available on GitHub at https://github.com/marinegenomicslab/FloridaBass_EpigeneticAgeing and on Zenodo at https://doi.org/10.5281/zenodo.17542324. Raw NovaSeq reads are available in the NCBI SRA (Bioproject PRJNA1301303).
